# Stem Cells: Microenvironment, Micro/Nanotechnology, and Application

**DOI:** 10.1155/2015/398510

**Published:** 2015-06-10

**Authors:** Hua Liu, Zhiyong Zhang, Wei Seong Toh, Kee Woei Ng, Shilpa Sant, António Salgado

**Affiliations:** ^1^Centre for Stem Cells and Tissue Engineering, School of Medicine, Zhejiang University, Hangzhou, Zhejiang 310058, China; ^2^Key Laboratory of Tissue Engineering and Regenerative Medicine of Zhejiang Province, Zhejiang University, Hangzhou, Zhejiang 310058, China; ^3^Department of Plastic and Reconstructive Surgery, Shanghai Ninth People's Hospital, School of Medicine, Shanghai Jiaotong University, Shanghai 200240, China; ^4^National Tissue Engineering Center of China, Shanghai Key Laboratory of Tissue Engineering, Shanghai 200240, China; ^5^Faculty of Dentistry, National University of Singapore, 11 Lower Kent Ridge Road, Singapore 119083; ^6^Tissue Engineering Program, Life Sciences Institute, National University of Singapore, 27 Medical Drive, Singapore 117510; ^7^School of Materials Science and Engineering, Nanyang Technological University, Singapore 639798; ^8^Department of Pharmaceutical Sciences, School of Pharmacy, Department of Bioengineering, Swanson School of Engineering, University of Pittsburgh, Pittsburgh, PA 15261, USA; ^9^McGowan Institute for Regenerative Medicine, University of Pittsburgh, Pittsburgh, PA 15219, USA; ^10^Life and Health Sciences Research Institute (ICVS), School of Health Sciences, University of Minho, Campus de Gualtar, 4710-057 Braga, Portugal

Hard tissue damage and loss caused by mechanical trauma, degenerative diseases, infections, tumors, and other diseases exert a profound negative impact on patients' quality of life and impose a heavy social and economic burden. Currently, tissue engineering is believed to be a promising approach to recover the structural integrity and function of these damaged or diseased hard tissues.

The 3 major elements of tissue engineered constructs are the seeded cells, the scaffolds, and the microenvironment. Seeded cells are considered to be the main component for exerting biological functions. Over the last decade, mesenchymal stem cells (MSCs) have been intensively studied as an ideal cell source for tissue engineering applications. Additionally, emerging biomaterial- and micro/nanotechnology-based platforms have advanced our understanding of the underlying mechanisms that determine the microenvironmental regulation of stem cell fate and functions, including self-renewal, proliferation, differentiation, and immune functions. In this special issue, work by P. Hartrianti et al. revealed that nanosized human keratin globules coated on tissue culture polystyrene could effectively enrich human MSCs (hMSCs)* ex vivo*. Due to the abundance, ready availability, and human origin of hair keratin, this material may provide a donor-customized microenvironment for hMSCs expansion* in vitro*.

Being one of the two major hard tissues in the human body, teeth require a wider range of seed cell sources for regeneration compared to bone, that is, stem cells derived from various dental tissues besides bone marrow and embryonic stem cells, such as dental pulp, dental papilla, periodontal ligament, dental follicle, apical papilla, and odontogenic epithelium. However, the limited availability of these seed cells severely limits their application. This special issue contains a paper by Y. Chen et al. that reports odontogenesis of human umbilical cord MSCs. Both the* in vitro* and* in vivo* data demonstrated the odontogenic potential of these cells. Another interesting paper by Q. Lu et al. in the dental field within this special issue showed that odontogenesis of dental pulp stem cells can be tuned by varying the crosslinking of polyethylene glycol-fibrinogen (PF) hydrogel on which the cells were seeded. A higher degree of mineralization of dental pulp stem cells was achieved with a more highly crosslinked PF hydrogel.

The regeneration of bone, another major hard tissue, has also been studied extensively. However, most previous studies focused on osteogenic efficiency by combining various MSCs with various bioscaffolds. It is important to understand the mechanisms behind the regeneration efficacy observed with different MSC-bioscaffold combinations, so as to optimize the mimicry of extracellular matrix-like environment. In this special issue, X. Zhang et al. showed that gelatin/*β*-TCP nanofibers promoted bone regeneration by activating calcium-sensing receptor signaling. Besides osteogenic differentiation of hMSCs, the local inflammatory microenvironment of cell grafts also plays an important role in influencing the efficacy of bone regeneration. D. Li et al. studied the immunoregulatory effects of hMSCs from ankylosing spondylitis patients, which could be enhanced by pretreatment with all-transretinoic acid. This could provide a new strategy to improve the efficacy of MSC-based therapy for ankylosing spondylitis.

As already mentioned, newly emerging biomaterials have advanced our understanding of the underlying mechanistic principles that govern the microenvironmental regulation of stem cell fate and function. Graphene is a revolutionary material, which was discovered by Nobel Laureates Geim and Novoselov in 2004. It has several properties that are advantageous for bone regeneration such as good electrical conductivity at room temperature, transparency, flexibility, and high mechanical strength. It can also provide a large surface area with ease of functionalization through attachment of various biomolecules. In this special issue, N. Dubey et al. intensively reviewed its characteristics, modifications, and potential applications in bone regeneration.

Nerves are important components of most human tissues including teeth and bone, which has the ability to orchestrate tissue remodeling and reorganization. In this special issue, R. C. Assunção-Silva et al. reviewed the research progress of 4 different stem cell types (embryonic stem cells, induced pluripotent stem cells, neural stem cells, and hMSCs) and 2 glial cell types (olfactory ensheathing cells and Schwann cells) in cell-based therapy for spinal cord injury (SCI). Amongst various materials, authors looked deeply into the roles of 6 natural-based hydrogels (alginate, agarose, collagen, fibrin, chitosan, and gellan-gum), 4 synthetic hydrogels (poly(lactic acid), poly(lactic-coglycolic acid), methacrylate, and poly(ethylene glycol)), and self-assembled peptides for SCI treatment. Based on the available knowledge, it was realized that cell transplantation by itself is inadequate for promoting tissue remodeling and axonal regeneration across dense glial scars. Scaffolds play a bridging role in this situation and provide a three-dimensional environment for the regenerating axons. Additionally, it is suggested that both drug delivery and tissue engineering are required for optimal nerve regeneration.

We hope that the readers will gain in-depth knowledge of various stem cell sources and biomaterials and their interactions in tissue regeneration through comprehensive reviews and research articles presented in this special issue. Most importantly, we also hope that this special issue will stimulate innovative research on the important questions to be resolved in future studies such as optimization of microenvironment to achieve sufficient number and stable quality of seed cells, rigorous evaluation of novel materials in tissue repair/regeneration, and the construction of functional tissues through innervation and vascularization.

*Hua Liu* *Hua Liu* *Zhiyong Zhang* *Zhiyong Zhang* *Wei Seong Toh* *Wei Seong Toh* *Kee Woei Ng* *Kee Woei Ng* *Shilpa Sant* *Shilpa Sant* *António Salgado* *António Salgado*

